# Interactions between 2-Cys peroxiredoxins and ascorbate in autophagosome formation during the heat stress response in *Solanum lycopersicum*

**DOI:** 10.1093/jxb/erw013

**Published:** 2016-01-31

**Authors:** Fei Cheng, Ling-Ling Yin, Jie Zhou, Xiao-Jian Xia, Kai Shi, Jing-Quan Yu, Yan-Hong Zhou, Christine Helen Foyer

**Affiliations:** ^1^Department of Horticulture, Zijingang Campus, Zhejiang University, Yuhangtang Road 866, Hangzhou, 310058, P.R. China; ^2^Key Laboratory of Horticultural Plant Biology, Ministry of Education/College of Horticulture and Forestry Sciences, Huazhong Agricultural University, Wuhan, 430070, P.R. China; ^3^Zhejiang Provincial Key Laboratory of Horticultural Plant Integrative Biology, 866 Yuhangtang Road, Hangzhou, 310058, P.R. China; ^4^Centre for Plant Sciences, Faculty of Biological Sciences, University of Leeds, Leeds, LS2 9JT, UK

**Keywords:** antioxidant metabolism, autophagy, 2-Cys peroxiredoxin, heat tolerance, oxidized protein, *Solanum*, *lycopersicum*, water-water cycle.

## Abstract

2-Cys peroxiredoxins (2-CPs) function in the removal of hydrogen peroxide and lipid peroxides but their precise roles in the induction of autophagy have not been characterized. Here we show that heat stress, which is known to induce oxidative stress, leads to the simultaneous accumulation of transcripts encoding 2-CPs and autophagy proteins, as well as autophagosomes, in tomato (*Solanum lycopersicum*) plants. Virus-induced gene silencing of the tomato peroxiredoxin genes *2-CP1*, *2-CP2*, and *2-CP1/2* resulted in an increased sensitivity of tomato plants to heat stress. Silencing *2-CP2* or *2-CP1/2* increased the levels of transcripts associated with ascorbate biosynthesis but had no effect on the glutathione pool in the absence of stress. However, the heat-induced accumulation of transcripts associated with the water-water cycle was compromised by the loss of 2-CP1/2 functions. The transcript levels of autophagy-related genes *ATG5* and *ATG7* were higher in plants with impaired 2-CP1/2 functions, and the formation of autophagosomes increased, together with an accumulation of oxidized and insoluble proteins. Silencing of *ATG5* or *ATG7* increased the levels of 2-CP transcripts and protein but decreased heat stress tolerance. These results demonstrate that 2-CPs fulfil a pivotal role in heat stress tolerance in tomato, via interactions with ascorbate-dependent pathways and autophagy.

## Highlight

2-Cys peroxiredoxins fulfil a pivotal role in heat stress tolerance in *Solanum lycopersicum* via interactions with ascorbate-dependent pathways and autophagy.

## Introduction

Plants are continuously exposed to changing environmental conditions. Plant functions can be compromised by exposure to excess light, extremes of temperature, limited nutrient or water availability, and pathogen/insect attack. An increased understanding of plant responses to these stresses, which disturb cellular homeostasis, is required to develop better strategies for crop improvement. All abiotic stress conditions result in an accumulation of reactive oxygen species (ROS), allowing oxidative signalling that leads to the changes in gene expression which underpin acclimation and adaptation to stress (Foyer and Noctor, 2005; Gill and Tuteja, 2010). Plants have evolved mechanisms that protect photosystem (PS) II and the electron transport system from photo-oxidative stress, including xanthophyll cycle-dependent energy dissipation, photorespiration, the D1 repair cycle, and cyclic electron flow around PSI. Moreover, ROS are scavenged by a network of enzymatic and non-enzymatic antioxidants, including ascorbate peroxidases (APXs) and peroxiredoxins (PRXs; Asada *et al.*, 1998; Noctor and Foyer, 1998, 2005; Awad *et al.*, 2015). Proteins that have become oxidized or otherwise modified as a result of stress can be removed by autophagy (Xiong *et al.*, 2007b; Slavikova *et al.*, 2008).

PRXs are a large family of thiol-based peroxidases that are widely distributed in all living organisms, from archaebacteria to mammals (Pulido *et al.*, 2009). In plants, PRXs have been organized into four distinct subclasses based on their genome sequences: 1-Cys PRX, 2-Cys PRX (2-CP), PRX II, and PRX Q (Dietz *et al.*, 2002; Rouhier and Jacquot, 2002). The enzymatic functions of PRXs are largely based on the presence of redox-active thiol groups (Petersson *et al.*, 2006). In the case of 2-CPs, which are found in chloroplasts, an intermolecular disulfide bridge is established between the catalytic Cys of one monomer and the Cys of a second monomer, leading to the formation of a homodimer (Horling *et al.*, 2002). Previously, we have reported that the disulfide bridge of 2-CPs in chloroplasts is reduced by thioredoxin-*m* (TRX-*m*) and thioredoxin-*f* (TRX-*f*) in tomato (*Solanum lycopersicum*) plants (Cheng *et al.*, 2014). PRXs catalyse the detoxification of a broad range of peroxides, including H_2_O_2_ and alkyl hydroperoxides. They are thus crucial to the antioxidative defences of plants that protect against the harmful effects of abiotic stress (Baier and Dietz, 1999; Tripathi *et al.*, 2009).

The water-water cycle has a key role in photoprotection. In the classic water-water cycle, superoxide radicals generated by the one-electron reduction of O_2_ by PSI are rapidly converted to H_2_O_2_ by Cu/Zn-superoxide dismutase (Cu/Zn-SOD) or Fe-SOD in the chloroplast (Asada, 1999). H_2_O_2_ is then detoxified and reduced to H_2_O by the ascorbate-glutathione cycle, which consists of APX, monodehydroascorbate reductase (MDAR), dehydroascorbate reductase (DHAR), and glutathione reductase (GR; Foyer and Noctor, 2005). Ascorbate and glutathione also function in combination with the ascorbate-glutathione cycle to reduce H_2_O_2_ and to dissipate excess excitation energy in chloroplasts (Foyer and Halliwell, 1976; Foyer and Noctor, 2005). *Arabidopsis thaliana* mutants lacking either the thylakoid *APX* (*tAPX*) or the stromal APX (*sAPX*) accumulated higher levels of H_2_O_2_ and oxidized proteins under photo-oxidative stress than the wild-type plants (Maruta *et al.*, 2010). However, Arabidopsis *sapx tapx* double mutants did not show large decreases in photoprotective capacity even under high light stress (Giacomelli *et al.*, 2007; Kangasjarvi *et al.*, 2008; Maruta *et al.*, 2010). Molecular genetic evidence supports the view that the plastid-localized 2-CPs work together with the tAPX to remove H_2_O_2_ from the chloroplasts (Awad *et al.*, 2015).

Exposure to stress conditions often results in an increased abundance of enzymatic and non-enzymatic antioxidants, including those involved in the water-water cycle (Foyer and Noctor, 2005). It is well established that heat stress results in a burst of ROS and that the heat-induced accumulation of ROS is required to elicit the heat shock response in gene expression. However, the roles of 2-CPs in mediating the heat shock response have not been characterized. Similarly, recent studies have shown that ROS induces the expression of genes associated with autophagy, suggesting that ROS are an important signal for the induction of the autophagy pathway (Xiong *et al.*, 2007a, 2007b; Perez-Perez *et al.*, 2012). Antisense suppression of a 2-CP in Arabidopsis specifically enhanced the levels of transcripts and activities of enzymes associated with ascorbate metabolism but had no effect on the glutathione pool (Baier *et al.*, 2000). The Arabidopsis *2cpa 2cpb* double mutants that are deficient in both of the chloroplast 2-Cys PRXs showed an increased sensitivity to high light stress and had lower photosynthetic efficiencies than either the wild type or *tapx* knockout mutants (Awad *et al.*, 2015). The plastid-localized 2-CPs therefore play a key role in the protection of photosynthesis against the harmful effects of excess illumination. Triple mutants that are deficient in both the plastid 2-Cys PRXs and the tAPX (*2cpa 2cpb tapx*) had significantly higher levels of oxidative stress-responsive transcripts than the *2cpa 2cpb* mutants or the wild-type plants (Awad *et al.*, 2015). The 2-CP and tAPX pathways therefore operate together in fulfilling water-water cycle functions and so modulate the H_2_O_2_ signal arising in the chloroplasts. It is therefore important to understand the relationships between 2-Cys PRXs and the ascorbate-glutathione cycle in other stress situations that involve significant ROS accumulation, such as heat stress, and in the oxidative stress-dependent induction of autophagy.

Autophagy is a highly conserved intracellular degradation system in eukaryotes for the removal and recycling of cytoplasmic components, including damaged proteins and organelles (Klionsky, 2005). Autophagy is induced in plants during abiotic stresses, such as oxidative, high salt, and osmotic stress conditions and infection by the necrotrophic fungal pathogen *Botrytis cinerea* (Slavikova *et al.*, 2008; Liu *et al.*, 2009; Lai *et al.*, 2011). Autophagy-defective RNAi-*AtATG18a* transgenic lines are hypersensitive to ROS, salt, and drought (Xiong et al., 2007a, 2007b; Liu *et al.*, 2009). Moreover, autophagy mutants exhibit enhanced susceptibility to the necrotrophic pathogens *B. cinerea* and *Alternaria brassicicola* (Lai *et al.*, 2011; Lenz *et al.*, 2011). Transgenic plants that are defective in the autophagy pathway (RNAi-*AtATG18a*) accumulate a higher level of oxidized proteins compared to controls (Xiong *et al.*, 2007a, 2007b). Autophagy is therefore a central pathway for the degradation of oxidized proteins during oxidative stress. Recently, we found that heat stress induces the expression of autophagy-related genes (*ATG*) and the accumulation of autophagosomes in tomato plants (Zhou *et al.*, 2014). Virus-induced gene silencing (VIGS) of several tomato *ATG* genes resulted in an increased sensitivity to heat stress, suggesting the involvement of autophagy in the heat stress response in tomato plants.

Many important crop species, such as tomato, are frequently faced with heat-induced oxidative stress during growth and fruit production. We therefore analysed the relationships between 2-CPs and autophagy in the heat stress tolerance response of tomato plants. VIGS was used to analyse the roles of the tomato 2-CP genes and ATG genes in the heat stress response. The data not only demonstrate that 2-CPs and autophagy play a critical role in heat tolerance in tomato, but also provide new insights into the relationships between 2-CPs and autophagy.

## Materials and Methods

### Plant materials, virus-induced gene silencing constructs and *Agrobacterium*-mediated virus infection

Tomato (*Solanum lycopersicum* L. cv. Condine Red) seeds were germinated in a growth medium composed of a mixture of peat and vermiculite (7:3, v:v) in trays in a growth chamber. When the first true leaf was fully expanded, the seedlings were transplanted into plastic pots (15cm diameter and 15cm depth, one seedling per pot) containing the same medium and were watered daily with Hoagland’s nutrient solution. The growth conditions were as follows: a 14-h photoperiod, temperature of 25/20°C (day/night) and photosynthetic photon flux density (PPFD) maintained at 200 µmol m^−2^ s^−1^.

To specifically silence *2-CP1*, *2-CP2*, *ATG5*, and *ATG7*, a 200–500bp section of the 3′ untranslated region was PCR-amplified with the primers listed in Supplementary Table S1, available at *JXB* online (The Tomato Genome Consortium 2012), and cloned into the XhoI-SacI or EcoRI-XhoI site of pTRV2. For the co-silencing of *2-CP1/2*, a 531bp fragment of *2-CP1* (nucleotides 447–977) was PCR-amplified using the forward primer CGGCGCTCGAGGAATTCATCAAGGTTAAAT and the reverse primer GGCGCGAGCTCTTATATGGATGCAAAGTAC; the primers contained XhoI and SacI restriction sites. The PCR fragment was inserted into the XhoI-SacI site within the multiple cloning region of the pTRV2 vector. The pTRV2-*2-CP1*, pTRV2-*2-CP2*, co-silencing pTRV2-*2-CP1/2,* pTRV2-*ATG5*, and pTRV2-*ATG7* VIGS constructs were confirmed by sequencing and then transformed into *Agrobacterium tumefaciens* strain GV3101.

*Agrobacterium*-mediated virus infection was performed as previously described (Ekengren *et al.*, 2003). An *Agrobacterium* culture carrying an empty pTRV2 vector was also infiltrated into a set of plants that were used as a control. The inoculated plants were maintained at 20–22°C in a growth chamber with a 14-h day length. After approximately 4 weeks, quantitative real-time PCR (qRT-PCR) was performed to determine the gene silencing efficiency before the plants were used in assays. The expression of *2-CP1*, *2-CP2*, *ATG5*, and *ATG7* in pTRV-*2-CP1*, pTRV-*2-CP2*, pTRV-*2-CP1/2*, pTRV-*ATG5*, and pTRV-*ATG7* plants is shown in Supplementary Fig. S1A, B, available at *JXB* online.

### Experimental design

There were three experiments in this study. In experiment I, plants at the six-leaf stage were placed at 22°C and 45°C in growth chambers (ConvironE15; Conviron, Manitoba, Canada) with 200 μmol m^−2^ s^−1^ PPFD for 7h. The heat stress treatment used in these studies did not lead to the death of any of the plants. Leaf samples were harvested at different time points from heated or unheated tomato plants, then frozen immediately in liquid nitrogen and stored at –80°C prior to gene expression and 2-CP redox status analyses. In experiment II and III, 2-CP and ATG VIGS plants at the six-leaf stage, respectively, were placed at 22°C and 45°C with 200 μmol m^−2^ s^−1^ PPFD in growth chambers for 7h and then immediately analysed for electrolyte leakage or maximum quantum yield of PSII (*F*v/*F*m). Leaf samples were harvested at different time points from heated or unheated tomato plants, then frozen immediately in liquid nitrogen and stored at –80°C prior to gene expression and protein analyses and antioxidant assays.

### Analysis of heat stress tolerance

Electrolyte leakage in the leaves was determined as previously described (Hong *et al.*, 2003). *F*v/*F*m was measured with IMAGING-PAM (IMAG-MAXI; Heinz Walz, Effeltrich, Germany) after the whole plants were dark-adapted for 30min. Minimal fluorescence (*F*o) was measured during the weak measuring pulses and maximal fluorescence (*F*m) was measured by an 0.8-s pulse of light at 4000 µmol m^−2^ s^−1^. *F*v/*F*m was then calculated as (*F*m − *F*o)/*F*m. *Fv/Fm* were determined using the whole area of the fifth leaf from the bottom. The light-saturated rate of CO_2_ assimilation (*A*_sat_) was measured with an open gas exchange system (LI-6400; LI-COR, Inc., Lincoln, NE, USA) on the fifth leaf of each plant under a CO_2_ concentration of 380 µmol mol^−1^, a saturating PPFD of 1500 µmol m^−2^ s^−1^, a leaf temperature of 25±1.5°C and a relative air humidity of 80–90%.

### Antioxidant assays

For antioxidant enzyme assays, leaf tissues (0.3g) were ground with a 2mL ice-cold buffer containing 50mM phosphate-buffered saline (pH 7.8), 0.2mM EDTA, 2mM L-ascorbic acid, and 2% (w/v) polyvinylpyrrolidone. Homogenates were centrifuged at 12 000 *g* for 20min, and the resulting supernatants were used to determine enzyme activity. The activity of SOD, catalase (CAT), APX, MDAR, DHAR, and GR were measured following the previously described protocol (Xia *et al.*, 2009). All spectrophotometric analyses were conducted on a Shimadzu UV-2410PC spectrophotometer (Shimadzu, Kyoto, Japan). Reduced ascorbate (AsA), dehydroascorbate (DHA), reduced glutathione (GSH), and oxidized glutathione (GSSG) content was measured as previously described (Jiang *et al.*, 2013).

### Total RNA extraction and gene expression analysis

Total RNA was isolated from tomato leaves using the TRIZOL reagent (Sangon, Shanghai, China) according to the manufacturer’s instructions. The cDNA template for qRT-PCR was synthesized from 2 µg of total RNA using a ReverTra Ace qPCR RT Kit (Toyobo, Osaka, Japan). Gene-specific primers were designed based on their cDNA sequences and employed for amplification, as described in Supplementary Table S2, available at *JXB* online. For the qRT-PCR analysis, we amplified PCR products in triplicate using iQ SYBR Green SuperMix (Bio-Rad, Hercules, CA, USA) in 25 µL qRT-PCR assays. The PCR was performed using an iCycler iQ 96-well real-time PCR Detection System (Bio-Rad, Hercules, CA, USA), and the cycling conditions consisted of denaturation at 95°C for 3min, followed by 40 cycles of denaturation at 95°C for 30s, annealing at 58°C for 30s, and extension at 72°C for 30s. The tomato *actin7* gene was used as an internal control. The relative gene expression was calculated as described by Livak and Schmittgen (2001).

### Non-reducing SDS-PAGE, separation of soluble and insoluble proteins, and western blotting

Total, soluble, and insoluble proteins from tomato leaves were assayed before and after heat treatment, as previously described (Zhou *et al.*, 2013). The concentrations of total, soluble, and insoluble proteins were determined using a protein assay kit (Bio-Rad), and BSA was used as a standard. Oxidized proteins from the soluble protein fraction were detected using an OxyBlot protein oxidation detection kit (Chemicon International, Temecula, CA, USA) according to the manufacturer’s instructions. The total, dimer forms, and monomer forms of 2-CPs were determined by non-reducing SDS-PAGE and western blot analysis as described previously (Cheng *et al.*, 2014). For the total protein abundance analysis of 2-CPs, β-mercaptoethanol was applied as the reducing agent in the protein extraction solution and protein samples were boiled before loading onto SDS-PAGE, in which only the monomer form could be detected on a western blot image. For the determination of dimer and monomer forms of 2-CPs, β-mercaptoethanol and boiling were omitted (Muthuramalingam *et al.*, 2010). 2-CPs were detected with a polyclonal antibody against 2-CysP (AbP80255-A-SE; Beijing Protein Innovation, Beijing, China). After incubation with a horseradish peroxidase-linked secondary antibody (Cell Signaling Technology, Boston, MA, USA), the antigen-antibody complexes were detected using an enhanced chemiluminescence kit (Perkin Elmer, Wellesley, MA, USA) according to the manufacturer’s instructions.

### Detection of autophagosome structures

For visualization of autophagosomes, tomato leaves were vacuum-infiltrated with 500 µM of the fluorescence dye monodansylcadaverine (MDC) (Sigma-Aldrich, St. Louis, MO, USA) at 1h after heat stress and kept for an additional hour in darkness before visualization (Xiong *et al.*, 2007b; Wang *et al.*, 2013). MDC-indicative autophagic structures were detected by a confocal laser scanning microscope (Leica TCS SL; Leica Microsystems, Wetzlar, Germany), excited by a wavelength of 405nm and detected at 400–580nm. Chloroplast autofluorescence was excited at 453nm and detected at 580–695nm.

To visualize autophagic structures at the subcellular level, excised tomato leaves were immediately cut and fixed according to the method described by Chen *et al.* (2009) and Wang *et al.* (2013). Sections were then examined using a TEM (H7650, Hitachi, Tokyo, Japan) at an accelerating voltage of 75kV.

### Statistical analysis

The experimental design was a completely randomized block design with four replicates. Each replicate contained at least 10 plants. Statistical analysis of the bioassays was performed using the SAS statistical package. The differences between the treatment means were separated using Tukey’s test at a level of *P* < 0.05.

## Results

### 2-CPs expression in response to heat stress in tomato

A database (Tomato Genome Sequencing Project) search based on sequence similarity with the predicted chloroplast 2-CPs of *A. thaliana* indicated two chloroplast 2-CP nucleotide sequences in *S. lycopersicum*: *2-CP1* (Solyc10g082030) and *2*-*CP2* (Solyc01g007740). A phylogenetic tree built from the alignment of the two proteins showed high similarity to the sequences of At2-CPA and At2-CPB, suggesting that they are orthologs of 2-CPA and 2-CPB in Arabidopsis (see Supplementary Fig. S2 at *JXB* online).

**Fig. 1.**
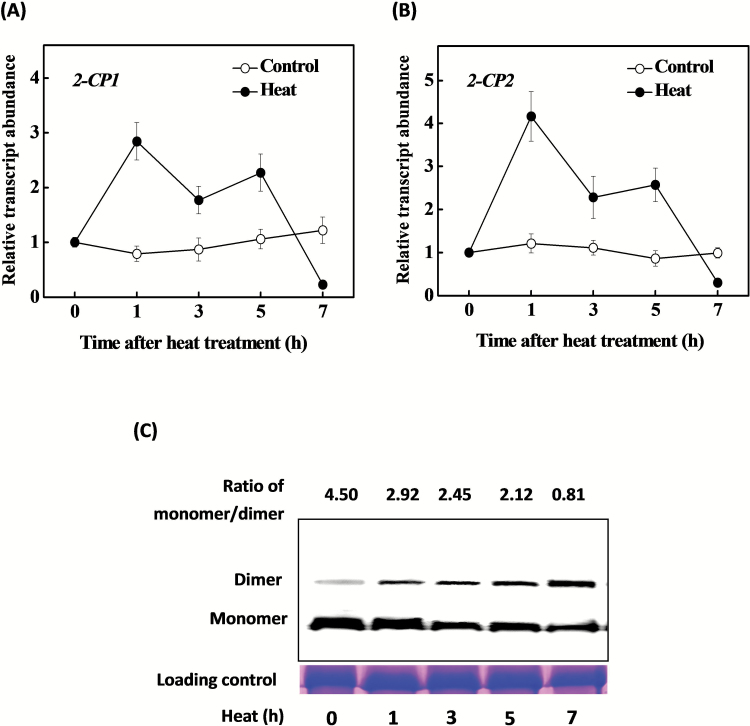
The gene expression (**A** and **B**) and redox state (**C**) of 2-CPs in response to heat at 45°C in tomato. Leaf samples were collected at the indicated times. The protein samples were separated by non-reducing SDS-PAGE and analysed by a western blot analysis with an anti-2-CP antibody. The ratio of 2-CP monomer to dimer was quantified by Quantity One software. The data are the means of four replicates with SD. Different letters indicate significant differences between the treatments according to Tukey’s test (*P* < 0.05).

 To determine whether 2-CPs are involved in the heat stress response in tomato, we first examined *2-CP1* and *2-CP2* expression during heat stress (Fig. 1A, B). While the levels of *2-CP1* and *2-CP2* transcripts were constant throughout the 7h period of the experiment in plants kept at 22°C, the abundance of transcripts encoding both 2-CP1 and 2-CP2 increased within 1h after the transfer to 45°C, and they remained high for 4h before declining to the same levels as in the controls maintained at 22°C. Western blot analysis showed that the 2-CPs monomer/dimer ratios were decreased from 4.50 at 0h to 2.92, 2.45, 2.12, and 0.81 at 1, 3, 5, and 7h respectively after the onset of the heat treatment (Fig. 1C).

### Heat tolerance is compromised in tomato plants lacking 2-CPs

The two tomato 2-CP genes, *2-CP1* and *2-CP2*, were silenced individually or in combination using a VIGS method. A transcript analysis of the leaflets in the middle of the fifth fully expanded leaf revealed that the *2-CP1* and *2-CP2* transcripts in the *2-CP1*-silenced (pTRV-*2-CP1*), *2-CP2*-silenced (pTRV-*2-CP2*), and *2-CP1* and *2-CP2*-co-silenced (pTRV-*2-CP1/2*) VIGS plants were reduced to ~21–35% of the levels in pTRV control plants (see Supplementary Fig. S1A at *JXB* online). The pTRV-*2-CP1*, pTRV-*2-CP2*, and pTRV-*2-CP1/2* plants grew more slowly than the pTRV plants. The co-silenced pTRV-*2-CP1/2* plants had the most pronounced slow-growth phenotype compared to the pTRV control plants (Supplementary Fig. 1B, C).

**Fig. 2.**
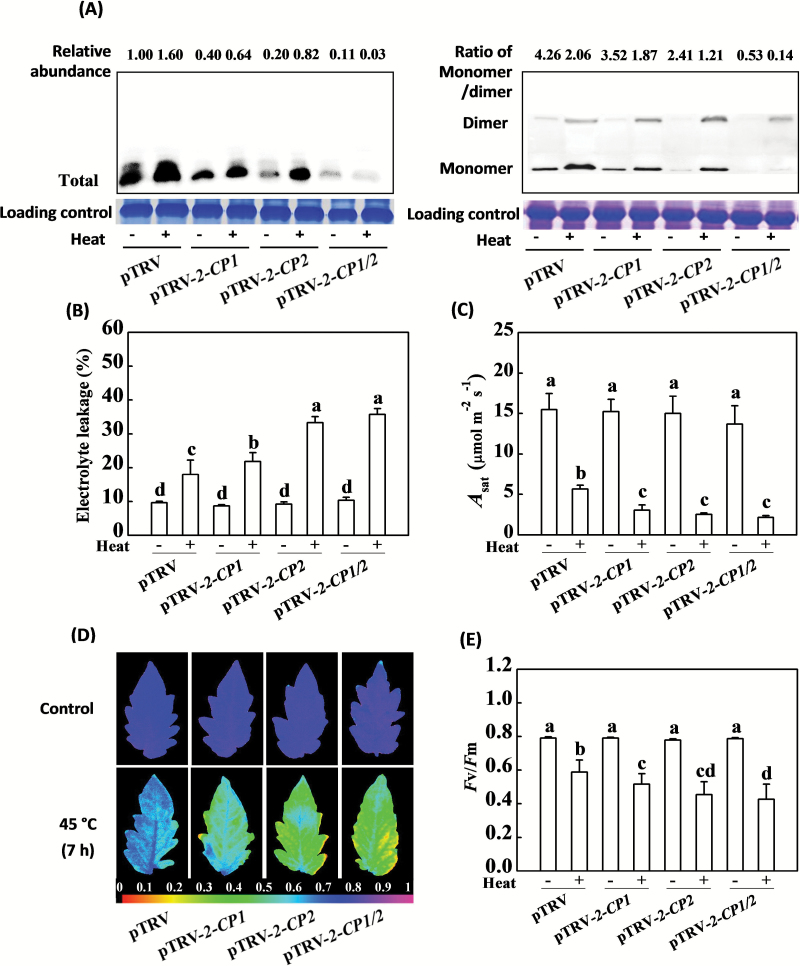
Compromised heat tolerance phenotypes of partially *2-CP*s-silenced plants. (**A**) The total protein abundance and redox state of 2-CPs in VIGS plants in response to heat stress. (**B**) Electrolyte leakage in response to heat stress. (**C**) Light saturated rate of CO_2_ assimilation (*A*_sat_) in response to heat stress. (**D**) Images of *F*v/*F*m in response to heat stress. (**E**) Average *F*v/*F*m values in response to heat stress. VIGS plants were placed at 22°C and 45°C in growth chambers for 7h and immediately measured for electrolyte leakage and *F*v/*F*m, and leaf samples were collected for 2-CPs protein abundance analysis. *A*_sat_ was measured after the plants were recovered at 25°C for 24h. The data are the means of four replicates with SD. Different letters indicate significant differences between the treatments according to Tukey’s test (*P* < 0.05).

Under optimal growth conditions, the silencing of either *2-CP1* or *2-CP2* resulted in lower 2-CP protein accumulation (Fig. 2A). Moreover, co-silencing of *2-CP1* and *2-CP2* (pTRV-*2-CP1/2*) almost completely abolished 2-CP protein accumulation (Fig. 2A). The exposure to heat stress resulted in increased 2-CP protein accumulation in the pTRV control, pTRV-*2-CP1*, and pTRV-*2-CP2* plants but 2-CP accumulation was negligible in the pTRV-*2-CP1/2* plants. Heat-induced 2-CP accumulation in the pTRV-*2-CP1*, pTRV-*2-CP2*, and pTRV-*2-CP1/2* plants was always lower than that in the pTRV plants. Meanwhile, a decrease in the 2-CPs monomer/dimer ratio was found in the gene-silenced plants after exposure to heat stress compared to the heat-treated pTRV plants (Fig. 2A).

The light-saturated rates of CO_2_ assimilation (*A*_sat_), electrolyte leakage, and the *F*v/*F*m values, that is, the maximum quantum yield of PSII, determined in fully expanded leaves in the *2-CP1*-, *2-CP2*-, and *2-CP1/2*-silenced plants were similar to those of the pTRV control plants in the absence of stress (Fig. 2B–E). After a 7h exposure to heat stress at 45°C, however, the electrolyte leakage values for the pTRV-*2-CP1*, pTRV-*2-CP2*, and pTRV-*2-CP1/2* plants were respectively 22%, 85%, and 99% higher than those of the pTRV plants (Fig. 2B). Similarly, heat stress caused a 15–30% decrease in the *F*v/*F*m ratios in the pTRV-*2-CP1*, pTRV-*2-CP2*, and pTRV-*2-CP1/2* plants relative to the pTRV control plants (Fig. 2D, E). Symptoms of heat-induced dehydration were visible only on the oldest leaves of the pTRV control plants. In contrast, most of the leaves on the pTRV-*2-CP1*, pTRV-*2-CP2*, and pTRV-*2-CP1/2* plants exhibited extensive wilting after the heat stress treatment (see Supplementary Fig. S1C). Similarly, *A*_sat_ was lower in the pTRV-*2-CP1*, pTRV-*2-CP2*, and pTRV-*2-CP1/2* leaves than those of the pTRV control plants after exposure to heat stress (Fig. 2C). These results show that partial silencing of *2-CP1*, *2-CP2*, and *2-CP1/2* increased the sensitivity of tomato plants to heat stress, suggesting that 2-CP1 and 2-CP2 play important roles in the heat stress response in tomato.

### 2-CPs-induced changes in antioxidant metabolism under heat stress

**Fig. 3.**
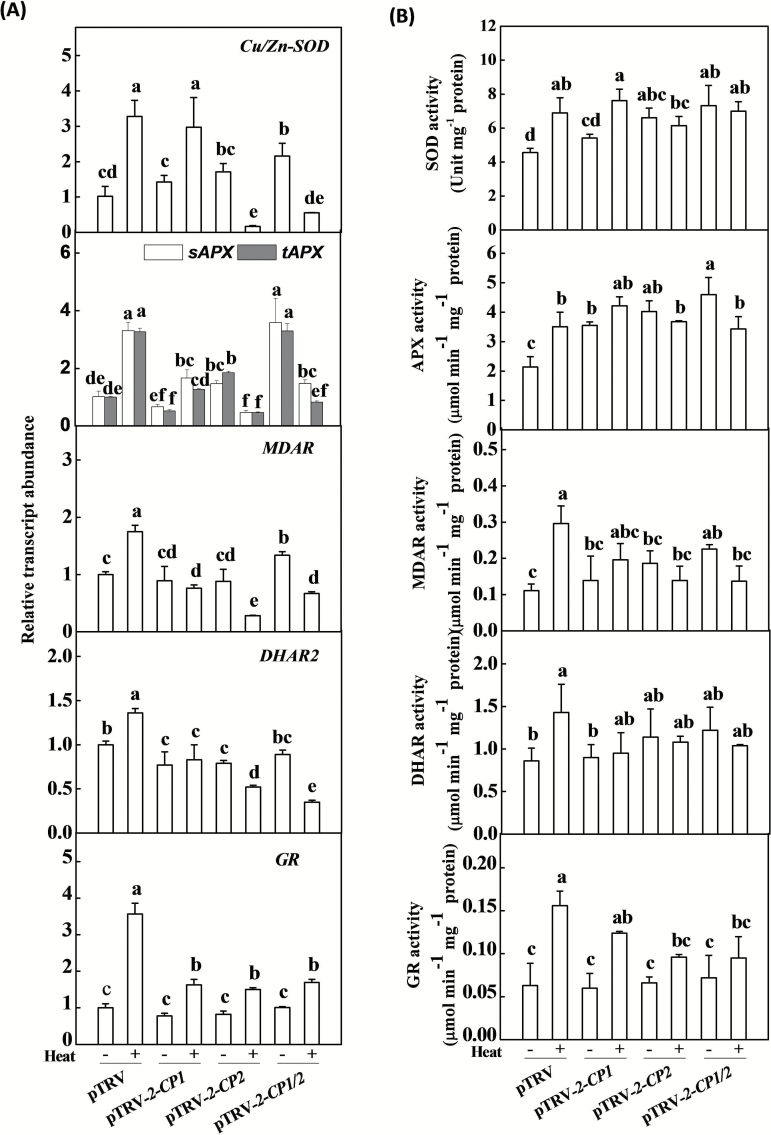
Changes in the levels of transcripts associated with the water-water cycle (**A**) and the activities of water-water-cycle-related enzymes (**B**) in response to heat stress in *2-CPs*-silenced plants. Leaf samples were collected after 7h of heat stress. The data are the means of four replicates with SDs. Different letters indicate significant differences between the treatments according to Tukey’s test (*P* < 0.05).

To examine whether 2-CPs are involved in the regulation of the antioxidative response in tomato, we examined changes in the transcript levels of seven antioxidant genes in the different VIGS plants in the absence or presence of heat stress. Of the transcripts measured in these studies, *Cu/Zn-SOD*, *sAPX*, *tAPX*, *MDAR*, *DHAR2*, and *GR* are involved in the scavenging of ROS in the chloroplasts, whilst *CAT2* plays key roles in the scavenging of cytosolic ROS and ROS generated by photorespiration in the peroxisomes, respectively (Fig. 3; Supplementary Fig. S3 at *JXB* online). While there were no significant changes in the *Cu/Zn-SOD*, *sAPX*, and *MDAR* transcripts in the pTRV*-2-CP1* or pTRV-*2-CP2* leaves in the absence of stress, these transcripts were increased in the pTRV*-2-CP1/2* plants relative to the pTRV control plants (Fig. 3A). The heat treatment led to significant increases in the levels of all of these transcripts, with increases ranging from 1.5-fold to 3.5-fold in the different pTRV plants. However, the silencing of *2-CP1*, *2-CP2*, and *2-CP1/2* resulted in different responses in antioxidant transcript levels to heat stress. In the pTRV-*2-CP1* plants, *Cu/Zn–SOD*, *sAPX*, *tAPX*, and *GR* transcript levels increased in response to heat stress relative to the pTRV control plants, whereas *MDAR* and *DHAR2* transcript levels did not (Fig. 3A). In contrast, heat stress in the pTRV-*2-CP2* and pTRV-*2-CP1/2* plants decreased the levels of all the measured antioxidant transcripts, with the exception of *GR* level, which was slightly increased (Fig. 3A). Moreover, the silencing of *2-CP1*, *2-CP2*, and *2-CP1/2* did not compromise heat-induced increases in *CAT2* transcripts (Supplementary Fig. S3A).

Silencing of *2-CP1*, *2-CP2*, and *2-CP1/2* led to different responses in the activities of SOD, APX, MDAR, DHAR, and GR. SOD activity was induced in pTRV-*2-CP2* and pTRV-*2-CP1/2* plants but not in TRV-*2-CP1* plants. In general, silencing of *2-CP1*, *2-CP2*, and *2-CP1/2* did not alter the activities of MDAR, DHAR, or GR. The only exception was the activity of MDAR in TRV-*2-CP1/2* plants, which was increased. Interestingly, APX activity was significantly higher as a result of silencing *2-CP1*, *2-CP2*, or *2-CP1/2* (Fig. 3B). Heat treatment resulted in significant increases in the activities of SOD, APX, MDAR, DHAR, GR, and CAT in the pTRV control plants (Fig. 3B; Supplementary Fig. S3B). This finding is consistent with the observed changes in the transcripts encoding the corresponding antioxidant enzymes (Fig. 3A). The heat-induced increases in the activities of these enzymes were largely compromised in the pTRV-*2-CP1*, pTRV-*2-CP2*, and pTRV-*2-CP1/2* plants relative to the pTRV controls (Fig. 3B; Supplementary Fig. S3B).

**Fig. 4.**
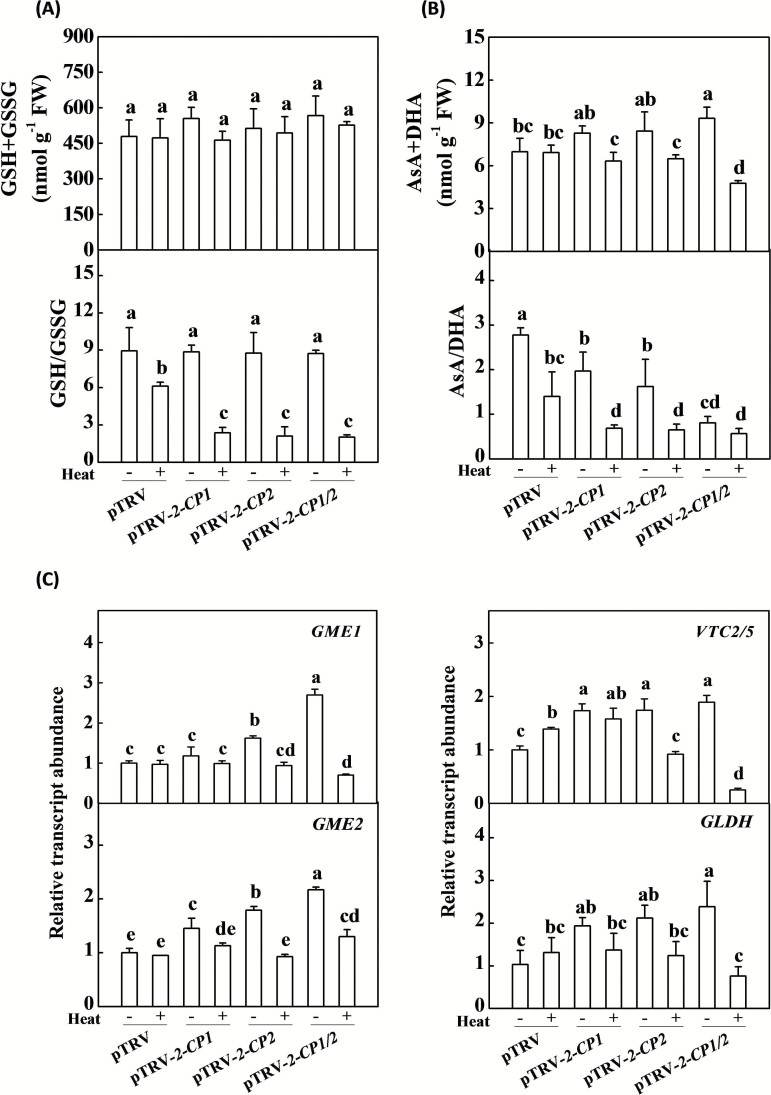
Changes in glutathione and ascorbate homeostasis and relative expression of critical genes involved in ascorbate biosynthesis in response to heat stress in VIGS plants. (**A**) Changes in glutathione homeostasis. (**B**) Changes in ascorbate homeostasis. (**C**) Changes in the transcript of genes involved in ascorbate biosynthesis. Leaf samples were collected after 7h of heat stress. The data are the means of four replicates with SDs. Different letters indicate significant differences between the treatments according to Tukey’s test (*P* < 0.05).

The total glutathione pool (GSH + GSSG) and the GSH to GSSG ratios in the leaves of pTRV-*2-CP1*, pTRV-*2-CP2*, and pTRV-*2-CP1/2* plants were not significantly different from those of the pTRV control plants measured in the absence of stress (Fig. 4A). While the heat treatment resulted in only a small decrease in leaf GSH content, there was a significant increase in leaf GSSG content, leading to a substantial decrease in the leaf GSH to GSSG ratios of the heat-stressed pTRV plants (Fig. 4A; Supplementary Fig. S4A at *JXB* online). The heat induced decreases in leaf GSH to GSSG ratios were more pronounced in the *2-CP1*, *2-CP2*, and *2-CP1/2*-silenced plants than in the pTRV controls (Fig. 4A).

While there were no significant changes in the levels of the total ascorbate (AsA + DHA) pool size as a result of heat stress in pTRV control plants, there was a decrease in the leaf AsA contents and an increase in the leaf DHA (oxidized ascorbate) levels, leading to decreased AsA to DHA ratios (Fig. 4B; Supplementary Fig. S4B). In the absence of stress, the level of the ascorbate pool was higher in the pTRV-*2-CP1/2* leaves than in the pTRV control plants (Fig. 4B; Supplementary Fig. S4B). This increase was mostly attributed to higher levels of DHA in the pTRV-*2-CP1/2* leaves. The heat stress treatment led to significant decreases in the size of the leaf ascorbate (AsA + DHA) pool in the pTRV-*2-CP1*, pTRV-*2-CP2*, and pTRV-*2-CP1/2* leaves, which was accompanied by significant decreases in the AsA to DHA ratio (Fig. 4B; Supplementary Fig. S4B). Moreover, silencing of *2-CP1*, *2-CP2*, and *2-CP1/2* induced increases in the levels of *GDP-D-mannose 3’,5’-epimerase* (*GME2*), *GDP-L-galactose phosphorylase* (*VTC2/5*), and *L-galactono-1,4-lactone dehydrogenase* (*GLDH*) transcripts, which are involved in ascorbate biosynthesis in the absence of stress (Fig. 4C). *GME1* transcripts were higher only in pTRV-*2-CP2* and pTRV-*2-CP1/2* plants, not in pTRV-*2-CP1* plants. Exposure to heat stress led to significant increases in the levels of *VTC2/5* transcripts but had little effect on *GME1*, *GME2*, or *GLDH* transcripts in the pTRV control plants (Fig. 4C). In contrast, while heat stress decreased the levels of these transcripts in the pTRV-*2-CP2* and pTRV-*2-CP1/2* plants, it had little effect on these transcripts in pTRV-*2-CP1* leaves (Fig. 4C). These results suggest that 2-CPs influence the regulation of ascorbate biosynthesis and that impaired 2-CPs expression has a strong influence on cellular redox homeostasis in response to heat stress.

### 2-CPs influence the heat-induced oxidation of soluble proteins and accumulation of insoluble proteins

**Fig. 5.**
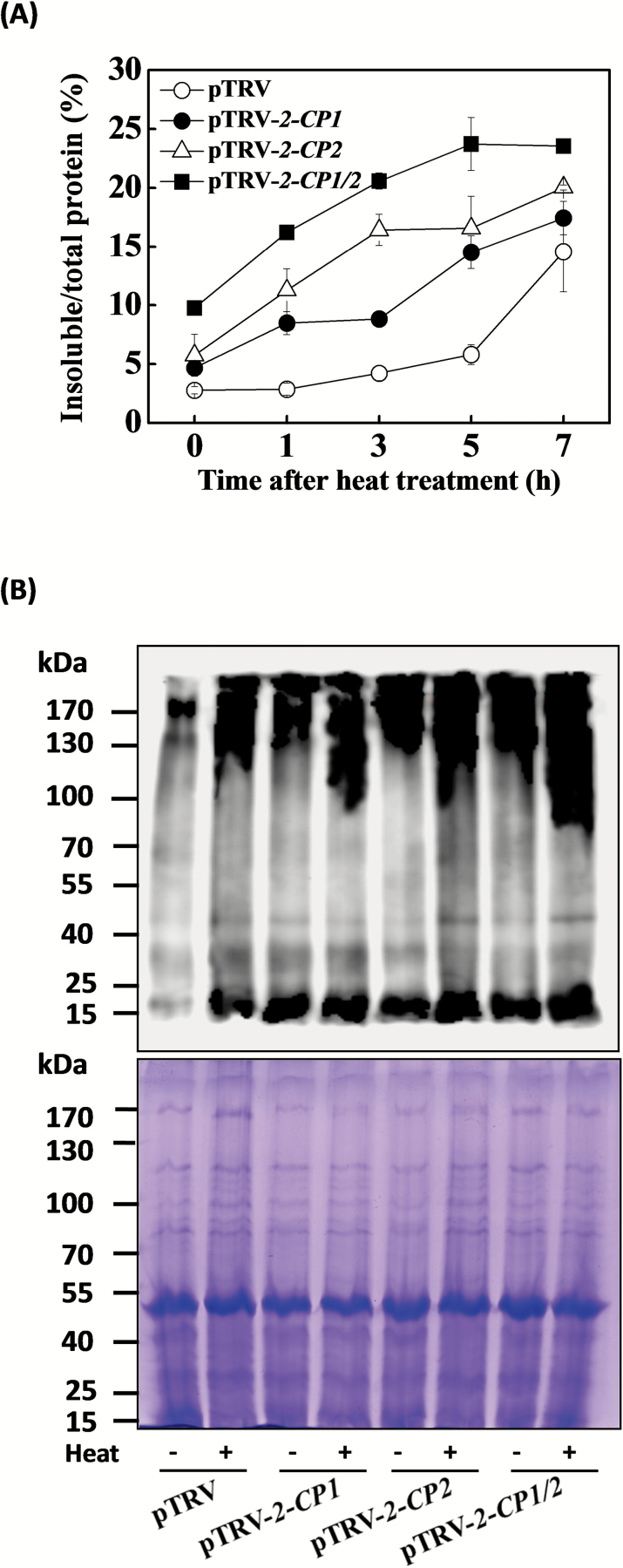
Enhanced oxidation of soluble proteins and increased accumulation of insoluble proteins in partially *2-CPs*-silenced plants under heat stress. (**A**) Time-course accumulation of insoluble proteins. Leaf tissues from VIGS plants were collected after the indicated time (h) at 45°C for the preparation of total, soluble, and insoluble proteins, as described in “Materials and Methods”. The percentages of insoluble proteins to total proteins were calculated by the determination of total proteins in the starting homogenates and insoluble proteins in the final pellets. The data are the means of four replicates with SDs. (**B**) Oxidation of soluble proteins. Coomassie Blue-stained gel of samples (lower panel) from part of the upper panel to demonstrate equal loading. Molecular size markers are indicated at the left. Leaf samples were collected after 7h of heat stress, and soluble proteins were isolated and derivatized by 2,4-dinitrophenol (DNP), followed by immunoblotting using an anti-DNP antibody.

The time-course accumulation of heat-induced oxidized and insoluble proteins was measured in the leaves of the pTRV, pTRV-*2-CP1*, pTRV-*2-CP2*, and pTRV-*2-CP1/2* plants (Fig. 5A). The percentage of insoluble protein relative to total proteins in the pTRV control plants did not increase until after 7h of heat stress treatment. In contrast, the ratio of insoluble to total proteins increased in the pTRV-*2-CP1*, pTRV-*2-CP2*, and pTRV-*2-CP1/2* leaves throughout the duration of exposure to heat stress. Moreover, the levels of oxidized protein were higher in the leaves of the gene-silenced plants relative to the pTRV controls in the absence of stress and following heat treatment (Fig. 5B). These findings suggest that 2-CPs fulfil important roles in the protection against protein oxidation and accumulation of insoluble proteins.

### Silencing of *2-CPs* promoted stress-induced autophagy under heat stress

**Fig. 6.**
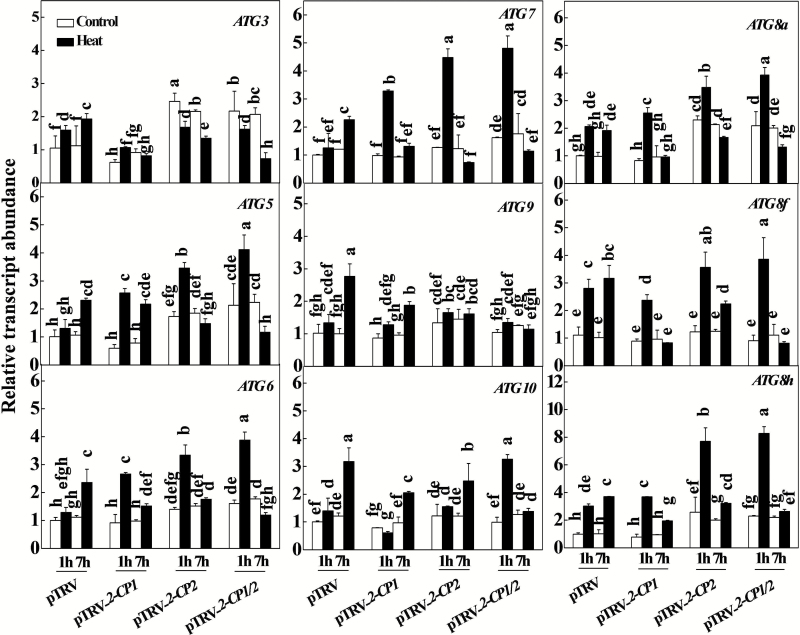
Induction of autophagy genes by heat stress in *2-CPs*-silenced plants. VIGS plants were placed at 45°C in growth chambers, and total RNA was isolated from leaf samples collected at the indicated times. The data are the means of four replicates with SDs. Different letters indicate significant differences between the treatments according to Tukey’s test (*P* < 0.05).

Autophagy is required for the degradation of oxidized proteins formed during natural and stress-induced senescence stress conditions. We therefore examined the expression patterns of the nine autophagy genes (*ATG3*, *ATG5*, *ATG6*, *ATG7*, *ATG 8a*, *ATG8f*, *ATG8h*, *ATG9*, and *ATG10*) in response to heat stress in the pTRV controls and the pTRV-*2-CP1*, pTRV-*2-CP2*, and pTRV-*2-CP1/2* plants. While the levels of these transcripts were not greatly changed in the *2-CP1*-, *2-CP2*-, and *2-CP1/2*-silenced plants compared to the pTRV plants in the absence of stress, the levels of *ATG3*, *ATG5*, *ATG6*, *ATG8a*, and *ATG8h* were significantly higher in the pTRV-*2-CP2* and pTRV-*2-CP1/2* plants than in the controls (Fig. 6). The accumulation of *ATG8a*, *ATG8f*, and *ATG8h* transcripts was increased after 1h of heat stress treatment in the pTRV controls, while others were significantly increased after 7h of heat stress (Fig. 6). The heat-induced accumulation of *ATG5*, *ATG6*, *ATG7*, and *ATG8h* transcripts occurred earlier in the pTRV-*2-CP1*, pTRV-*2-CP2*, and pTRV-*2-CP1/2* plants than in the controls (Fig. 6).

**Fig. 7.**
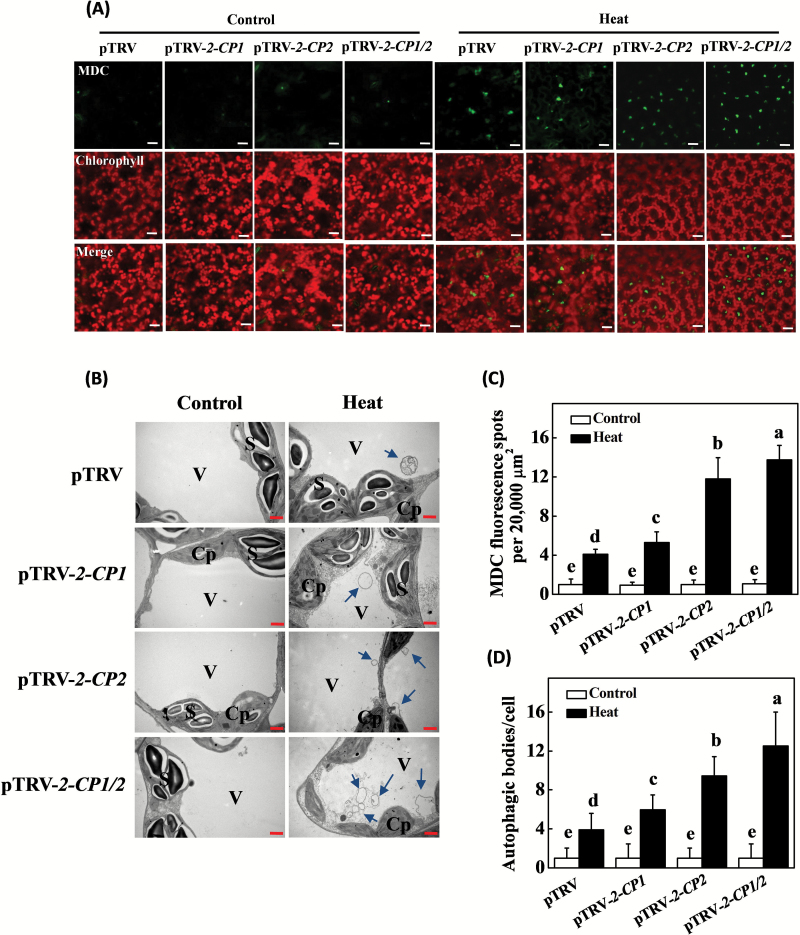
Detection of autophagosome structures. (**A**) MDC-stained autophagosomes in tomato leaves. The leaves were visualized by fluorescence confocal microscopy to allow the detection of autophagosome structures or chlorophyll-derived red fluorescence. The green channel images of MDC-derived fluorescence are superimposed with the red autofluorescence of chloroplasts. Bars = 25 µm. (**B**) Formation of autophagosomes in tomato leaves under heat stress using TEM. Cp, chloroplast; S, starch grain; V, vacuole; blue arrows, autophagosomes. Bars = 1 µm. (**C**) Numbers of punctate MDC fluorescence spots per 20 000 μm^2^ section are indicated. Means and SD were calculated from three experiments. (**D**) Numbers of autophagic structures in the mesophyll cells. Means and SD were calculated from four experiments, and each replicate was the average number of 100 mesophyll cells. Different letters indicate significant differences between the treatments according to Tukey’s test (*P* < 0.05). Leaf samples were taken at 1h after the heat stress.

Autophagosome formation was determined in tomato leaves from plants that had been exposed to 45°C for 1h. In the absence of stress, very few autophagosome structures were observed in the leaves of the pTRV controls or the pTRV-*2-CP1*, pTRV-*2-CP2*, and pTRV-*2-CP1/2* plants (Fig. 7). Exposure to heat stress resulted in an increase in the presence of autophagosomes in all plants. The silencing of *2-CP1*, *2-CP2*, and *2-CP1/2* led to a greater accumulation of heat-induced autophagosomes than was observed in the pTRV controls (Fig. 7). Heat stress therefore led to expression of the ATG genes in tomato leaves and to the formation of autophagosomes. These processes were observed earlier in the response to heat stress in pTRV-*2-CP1*, pTRV-*2-CP2*, and pTRV-*2-CP1/2* plants than in the controls (Figs 6 & 7). Meanwhile, we found less accumulation of starch in the chloroplasts of pTRV-*2-CP1*, pTRV-*2-CP2*, and pTRV-*2-CP1/2* leaves than in the controls (Fig.7B).

### Silencing of *ATG5* and *ATG7* impaired the heat tolerance and induced accumulation of 2-CPs

**Fig. 8.**
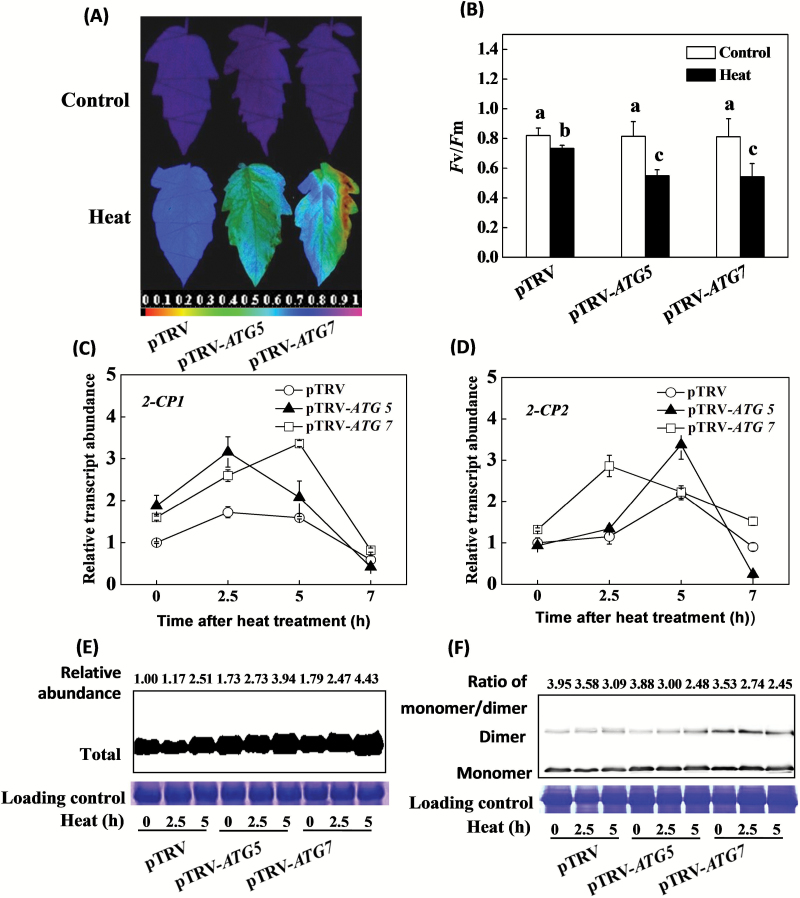
*ATG5* and *ATG7* silencing induced changes in heat tolerance, transcript levels of *2-CP1* and 2*-CP2*, and 2-CP accumulation in tomato plants. (**A**) Images of *F*v/*F*m in response to heat stress. (**B**) Average *F*v/*F*m values in response to heat stress. (**C**, **D**) The expression of *2-CP*s in response to heat stress. (**E**, **F**) The protein abundance of 2-CPs in response to heat stress. VIGS plants were placed at 22°C and 45°C in growth chambers for 7h and immediately measured for *F*v/*F*m. Leaf samples were collected at 0, 2.5, and 5h after heat stress for 2-CPs protein abundance analysis. The data are the means of four replicates with SDs. Different letters indicate significant differences between the treatments according to Tukey’s test (*P* < 0.05).

To explore the relationship between 2-CPs and autophagy further in relation to heat tolerance of tomato, *ATG5* and *ATG7* were silenced using the VIGS method. The pTRV-*ATG5* and pTRV-*ATG7* plants generated in this way showed an increased sensitivity to heat stress (Fig. 8A, B) as determined by the heat-stress induced decreases in *F*v/*F*m ratios. In addition, the heat-induced increases in the accumulation of *2-CP1*, *2-CP2*, and 2-CPs proteins were higher in the pTRV-*ATG5* and pTRV-*ATG7* plants than in the pTRV controls (Fig. 8C–F). Western blot analysis revealed that there was a more significant increase in the accumulation of dimer 2-CPs in pTRV-*ATG5* and, especially, pTRV-*ATG7* plants relative to the pTRV controls after exposure to heat stress. As a result, a larger decrease in the 2-CPs monomer to dimer ratio was found in response to heat stress in pTRV-*ATG5* and pTRV-*ATG7* plants than in the pTRV controls (Fig. 8F).

## Discussion

Although the functions of the chloroplast 2-CPs have been intensively studied (Baier and Dietz, 1999; Broin *et al.*, 2002; Awad *et al.*, 2015), the precise roles of these redox proteins in heat stress responses have not been fully characterized, particularly in relation to the regulation of autophagy and other antioxidants. Using a similar VIGS approach in *Nicotiana benthamiana*, a recent study showed that the loss of *2-Cys peroxiredoxin* family and *type-II peroxiredoxin B* proteins impaired the heat stress response through effects on AsA regeneration, the xanthophyll cycle, and downstream abscisic acid signalling (Vidigal *et al.*, 2015). The relationships between the 2-CPs and the ascorbate-mediated detoxification systems are particularly interesting because the chloroplast-localized 2-CPs and the tAPX have overlapping functions in H_2_O_2_ removal (Awad *et al.*, 2015). Moreover, while it is established that these antioxidant systems act synergistically to protect the photosynthetic apparatus against the negative effects of excess light (Awad *et al.*, 2015), relatively little is known about the co-operation and redundancy between these systems and autophagy in other abiotic stress responses. The data presented in this manuscript allow us to draw a number of conclusions, discussed below.

### 2-CPs are required for heat stress tolerance in tomato

Heat stress induced the expression of *2-CP1* and *2-CP2* and led to 2-CP protein accumulation in tomato. Interestingly, silencing *2-CP1* and *2-CP2* did not induce photoinhibition in the absence of the heat stress as observed in *2cpa* and *2cpb* mutants of Arabidopsis (Awad *et al.*, 2015). However, silencing of *2-CP1* and *2-CP2* resulted in an increased sensitivity to heat stress (Figs 1A, B and 2), confirming the role of these redox proteins in the heat stress response. The data presented here demonstrate that 2-CPs are required in an appropriate response to heat stress, as they are in plant responses to other environmental stresses (Horling *et al.*, 2002; Kim *et al.*, 2009). The abundance of 2-CP transcripts is increased by cold, salt, and oxidative (H_2_O_2_) stresses, as well as wounding in *A. thaliana*, *Vigna radiata* (mung bean), and *Oryza sativa* (rice) (Horling *et al.*, 2003; Cho *et al.*, 2012; Kim *et al.*, 2013). In addition, the expression of 2-CP genes is regulated by ascorbate (Horling *et al.*, 2003). Here we show that exposure to heat stress led to a significant increase in *2-CP1* and *2-CP2* transcripts within an hour of the imposition of the stress and this was followed by an increase in 2-CP protein accumulation (Fig. 1). A previous study showed that peroxide reduction by 2-CP in tomato chloroplasts was dependent on thioredoxins, especially *TRX-x*, *TRX-m1/4*, and *TRX-m2* (Cheng *et al.*, 2014). The subsequent decline in *2-CP1* and *2-CP2* transcripts after 5h of heat stress treatment (Fig. 1A, B) was accompanied by a decrease in the 2-CP monomer to dimer ratios in the tomato leaves (Fig. 1C). Taken together, these findings suggest that either the biosynthesis of 2-CPs or the regeneration of the reduced forms could not keep pace with chloroplast H_2_O_2_ production under heat stress.

The pTRV-*2-CP2* plants were more sensitive to heat stress than the pTRV-*2-CP1* plants (Figs 2B, 3, and 5A). Thus, even though 2-CP1 and 2-CP2 are 86% identical at the amino acid level, these proteins appear to play somewhat different roles in the heat stress response. Moreover, the higher insoluble to total protein ratios and lower AsA to DHA ratios observed in the pTRV-*2-CP1/2* plants compared to either the pTRV-*2-CP1* or pTRV-*2-CP2* plants suggests synergistic effects between *2-CP1* and *2-CP2* in the heat stress response (Figs 4B and 5A).

### Heat stress tolerance requires an interaction between 2-CP and the ascorbate-glutathione cycle in tomato

H_2_O_2_ is produced at high rates by photosynthesis, particularly under stress conditions (Noctor *et al.*, 2002). In the chloroplasts, 2-CPs act together with the ascorbate-glutathione cycle to remove H_2_O_2_ and protect the photosystems from photo-oxidative stress (Asada *et al.*, 1998; Foyer and Shigeoka, 2011; Awad *et al.*, 2015). Exposure to heat stress increased the abundance of transcripts encoding enzymes of the ascorbate-glutathione cycle and superoxide dismutase, as well as the activities of these enzymes in the pTRV control plants, as has been reported previously in tomato (Nie *et al.*, 2013). The data presented here provide further evidence of the interdependent functions of these antioxidant systems in the stress responses in tomato plants. These findings confirm the involvement of SOD and the ascorbate-glutathione cycle in the heat response in tomato plants.

Like other antioxidants, 2-CPs also fulfil signalling functions associated with H_2_O_2_ metabolism in chloroplasts (Dietz *et al.*, 2006). The levels of *Cu/Zn-SOD*, *sAPX*, *tAPX*, and *MDAR* transcripts were higher in the pTRV-*2-CP1/2* tomato plants in the absence of stress (Fig. 3A), in which the activities of SOD, APX, and MDAR were also increased relative to pTRV controls (Fig. 3B). These findings agree with earlier observations showing that the suppression of 2-CP functions in Arabidopsis resulted in an oxidation of the ascorbate pool, and led to increased expression and activities of sAPX, tAPX, and MDAR in the absence of stress (Baier *et al.*, 2000). Taken together, these results demonstrate the coordinated compensatory functions of the 2-CP and ascorbate-glutathione systems in water-water cycle activities. Moreover, the heat-induced increases in ascorbate-glutathione cycle-related transcripts and enzyme activities were largely compromised in the *2-CP*-silenced plants, resulting in substantial decreases in the AsA to DHA and the GSH to GSSG ratios (Figs 3 and 4A, B). All these results suggest that 2-CP-mediated oxidative signalling is required for the expression of the *SOD* and ascorbate-glutathione cycle-related genes in tomato. The data presented here also revealed the function similarity of 2-CPs in tomato and in Arabidopsis in response to heat and other stresses (Baier *et al.*, 2000; Foyer and Shigeoka, 2011; Awad *et al.*, 2015).

The pTRV-*2-CP1/2* plants showed a higher accumulation of leaf ascorbate than did the pTRV controls in the absence of stress (Fig. 4B). This finding is consistent with previous observations showing that loss of 2-CP function results in the expression of genes involved in ascorbate biosynthesis in Arabidopsis in the absence of stress (Baier *et al.*, 2000). The loss of 2-CP functions would place an additional burden on the ascorbate-glutathione pathways of H_2_O_2_ detoxification, leading to the observed decreases in the AsA to DHA and the GSH to GSSG ratios relative to the pTRV controls, as discussed previously (Dietz *et al.*, 2002). However, this compensatory action does not explain why the heat-stress-dependent induction of the expression of ascorbate biosynthesis genes was compromised in the pTRV-*2-CP1*, pTRV-*2-CP2*, and pTRV-*2-CP1/2* plants (Fig. 4B, C). One possibility suggested by these findings is that the 2-CP-dependent oxidative signalling pathway is required for the regulated expression of ascorbate biosynthesis genes.

### 2-CPs are required for the oxidative regulation of autophagy in the heat stress response in tomato

The data presented here show that the activities of 2-CPs attenuate protein misfolding and denaturation under heat stress. Loss of *2-CP1*, *2-CP2*, or *2-CP1/2* functions resulted in increased levels of oxidized soluble proteins that may aggregate into high molecular weight insoluble proteins (Fig. 5B). The lower levels of heat tolerance exhibited by the pTRV-*2-CP1*, pTRV-*2-CP2*, and pTRV-2-*CP1/2* plants were also associated with an increased accumulation of insoluble protein (Fig. 5A).

The efficient removal of misfolded and denatured proteins is required to prevent the cells from proteotoxic stress (Hightower, 1991). This can be achieved by autophagy, which is a major process involved in the dismantling of cellular structures during natural and stress-induced senescence (Slavikova *et al.*, 2008; Liu *et al.*, 2009; Liu and Bassham, 2010; Zhou *et al.*, 2013). Heat tolerance was suppressed in the *ATG5*- and *ATG7*-silenced tomato plants, which is in agreement with a previous study (Zhou *et al.*, 2014). The data presented here confirm that autophagy plays an important role in heat tolerance in tomato. The levels of *2-CP1* and *2-CP2* transcript and 2-CP protein were significantly higher in the *ATG5*- and *ATG7*-silenced plants that had been exposed to heat stress (Fig. 8C–F). These findings suggest that 2-CP functions are important in the regulation of autophagy, a finding that might be related to the transmission of ROS signals, which are known to induce autophagy in plants exposed to environmental stresses (Liu and Bassham, 2010). In these studies, the observed changes in ATG transcripts occurred in parallel to increases in the ratios of insoluble to total protein (Figs. 5A and 6). The levels of *ATG5* and *ATG7* transcripts increased more rapidly in the plants that were defective in 2-CP functions. Additionally, the *2-CP*-silenced plants had greater numbers of autophagosomes in line with the increased accumulation of oxidized proteins and production of insoluble protein (Figs 5–7). On the other hand, the *ATG5*- and *ATG7*-silenced plants had increased transcript levels and accumulation of 2-CPs in response to the heat stress (Figs. 8C–F), suggesting that 2-CPs and autophagy could function co-ordinately to prevent the cells from heat-induced oxidative damage in tomato. It is also worth noting that induction of autophagy was associated with less accumulation of starch in the chloroplasts of heat-treated *2-CP*-silenced plants, suggesting a potential role of 2-CPs in starch metabolism. This is in agreement with an earlier finding that autophagy induces rapid starch degradation in Arabidopsis (Wang and Liu, 2013)

In summary, the results of this study provide new links between the interactive 2-CPs and ascorbate-glutathione ROS-processing systems of chloroplasts. Evidence is provided to support the concept that both pathways function together in ROS detoxification during heat stress in tomato. Moreover, the data suggest that 2-CPs may also fulfil a signalling role linked to oxidant processing, the induction of autophagy, and the removal of oxidized proteins during heat stress.

## Supplementary Data

Supplementary data are available at *JXB* online.

Fig. S1. Relative mRNA abundance of *2-CP1*, *2-CP2*, *ATG5*, and *ATG7*, and phenotypes in VIGS plants.

Fig. S2. Phylogenetic tree of 2-Cys peroxiredoxins from *Solanum lycopersicum.*

Fig. S3. Relative mRNA abundance of *CAT2* and activity of CAT in *2-CP*-silenced plants after a heat stress.

Fig. S4. Changes in GSH, GSSG, AsA, and DHA content in *2-CP*-silenced plants after heat stress.

Table S1. PCR primers designed for vector construction.

Table S2. Gene-specific primers designed for qRT-PCR.

Supplementary Data
