# Supplementary material for: Interactions between 2-Cys peroxiredoxins and ascorbate in autophagosome formation during the heat stress response in Solanum lycopersicum
Source: J Exp Bot. 2016 Jan 31;67(6):1919–33. doi: 10.1093/jxb/erw013 (PMC4783371; doi:10.1093/jxb/erw013)
Supplement: Supplementary Data [file supp_67_6_1919__index.html]

Interactions between 2-Cys peroxiredoxins and ascorbate in autophagosome formation during the heat stress response in Solanum lycopersicum — Interactions between 2-Cys peroxiredoxins and ascorbate in autophagosome formation during the heat stress response in Solanum lycopersicum — Supplementary Data 

# Interactions between 2-Cys peroxiredoxins and ascorbate in autophagosome formation during the heat stress response in *Solanum lycopersicum*

## Supplementary Data

Data files

- supplementary\_figures\_S1\_S4\_Tables\_S1\_S2.pdf - Supplementary Data
