# Supplementary material for: Interactions between 2-Cys peroxiredoxins and ascorbate in autophagosome formation during the heat stress response in Solanum lycopersicum
Source: J Exp Bot. 2016 Jan 31;67(6):1919–33. doi: 10.1093/jxb/erw013 (PMC4783371; doi:10.1093/jxb/erw013)
Supplement: Supplementary Data [file supp_erw013_supplementary_figures_S1_S4_Tables_S1_S2.pdf]

Supplementary data

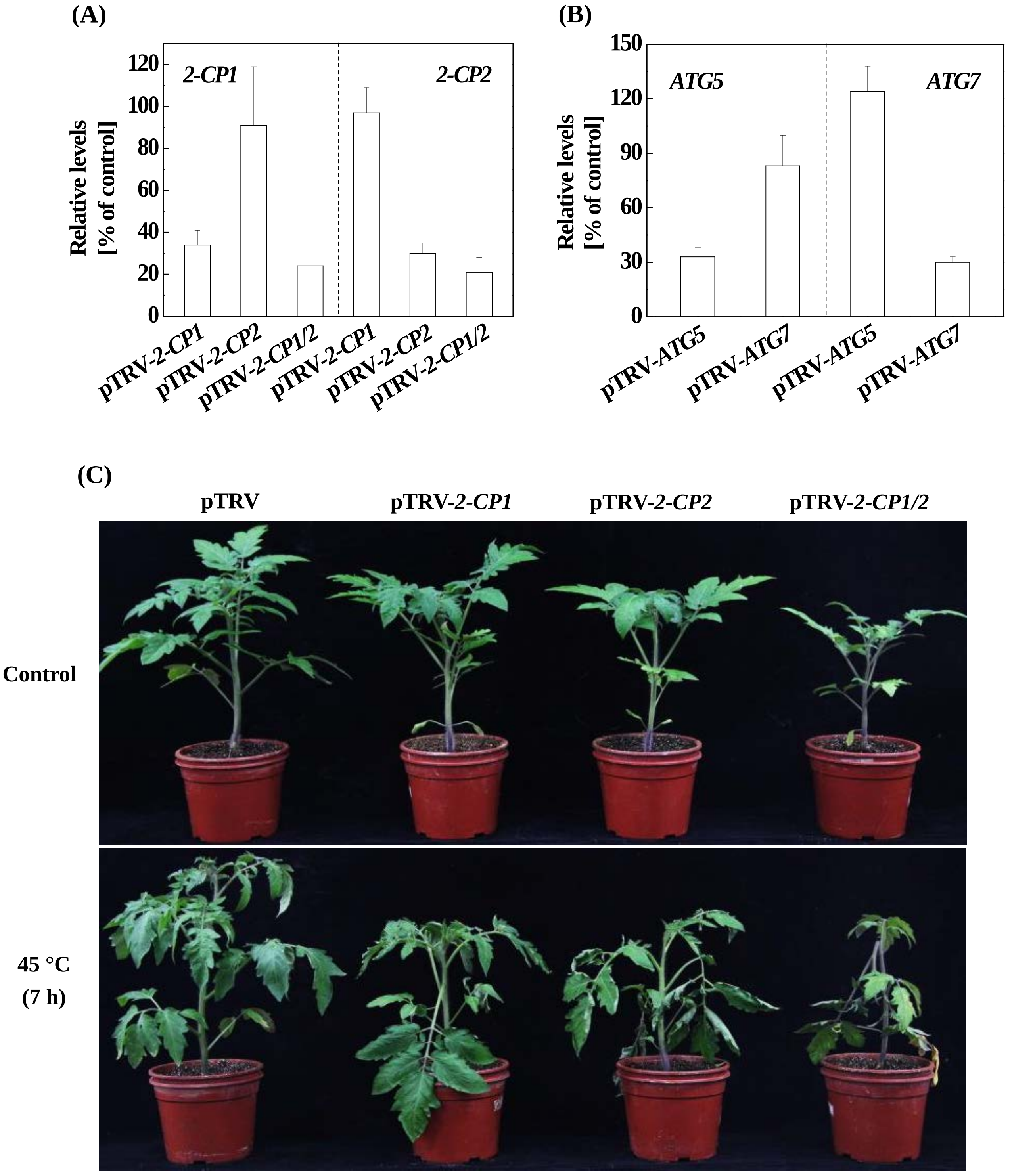

Figure S1. Relative mRNA abundance of *2-CP1*, *2-CP2*, *ATG5* and *ATG7,* and phenotypes in virus-induced gene silencing (VIGS) plants. (A) Relative mRNA abundance of *2-CP1* and *2-CP2* in virus-induced gene silencing (VIGS) plants. (B) Relative mRNA abundance of *ATG5* and *ATG7* in virus-induced gene silencing (VIGS) plants. Samples were taken at 30 d after *Agrobacterium*-infection. Data are the means of the 5th leaf of 10 silenced plants (±SD). The levels were expressed as percentages of the mean levels in control pTRV plants which were defined as 100 %. (C) Phenotypes of *2-CP*-silenced VIGS plants.

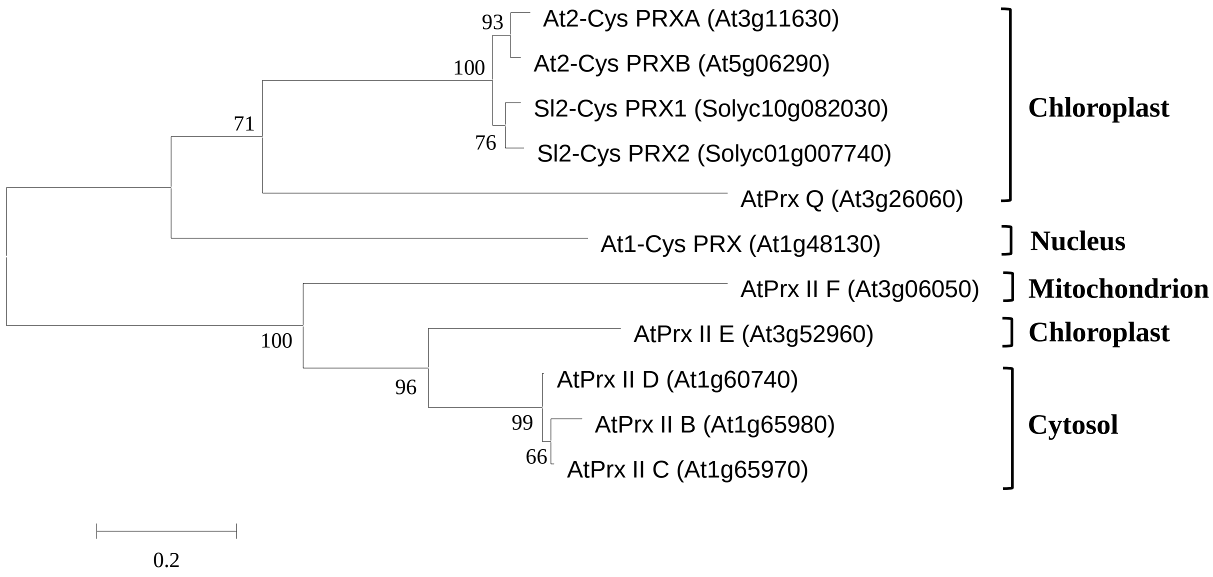

Figure S2. Phylogenetic tree of 2-Cys peroxiredoxins from *Solanum lycopersicum* (Sl) and those identified peroxiredoxins from *Arabidopsis* (At). The phylogenetic tree was constructed using MEGA 5 with the Neighbor–Joining method. Bootstrap values calculated from 1000 trials are shown at each node. The extent of divergence according to the scale (relative units) is indicated at the bottom. Predicted mature polypeptides lacking the putative transit peptide were employed for tree construction.

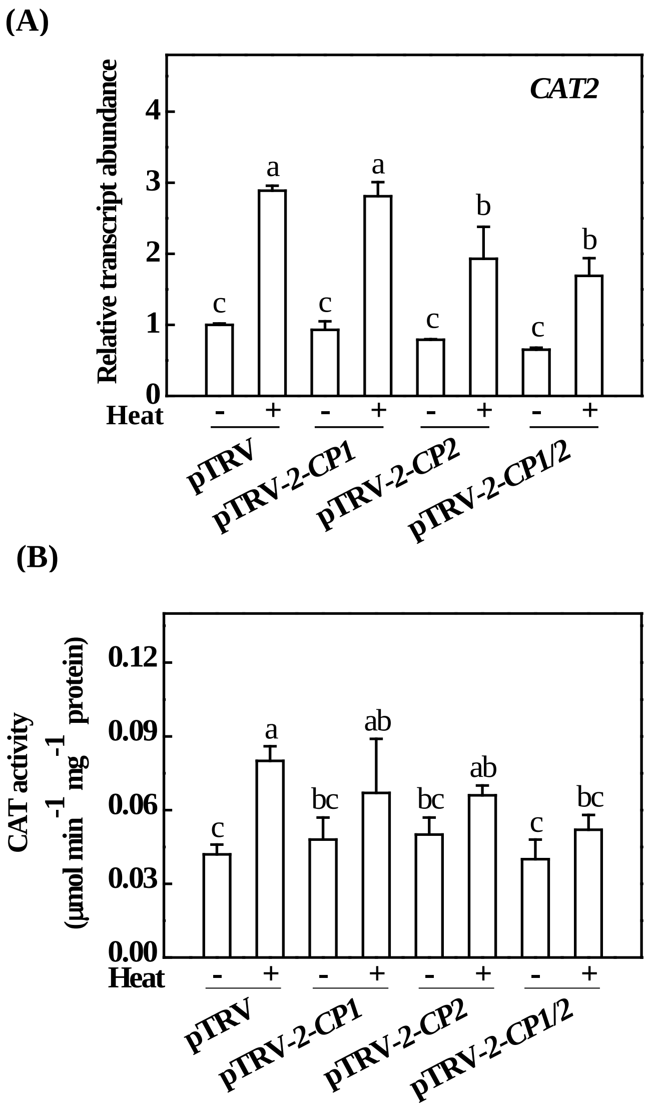

Figure S3. Relative mRNA abundance of *CAT2* (A) and activity of CAT (B) in *2-CP*-silenced plants after a heat stress.

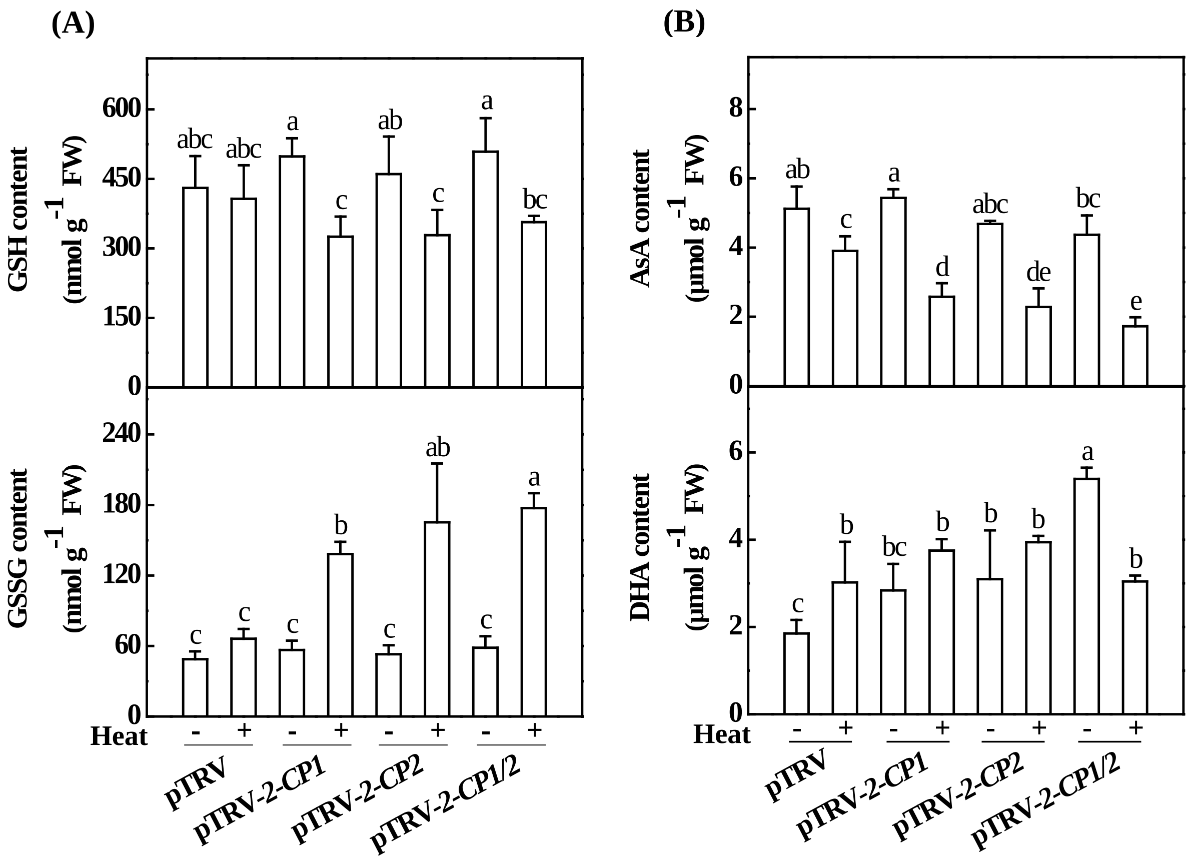

Figure S4. Changes in GSH, GSSG, AsA and DHA content in *2-CP*-silenced plants after a heat stress.

Table S1. PCR primers designed for vector construction.

Vector	Forward primer	Reverse primer
pTRV2-*2-CP1*	CGGCGCTCGAGGGAGATGAATTACATTGCT	GGCGCGAGCTCTTGGTACGTGCACTTTTGG
pTRV2-*2-CP2*	CGGCGCTCGAGGTCGATGAGATGATGGCTG	GGCGCGAGCTCTGAGACAAAAAGACTCAAT
pTRV2-*ATG5*	CGGAATTCAACGGCCTTGGAATCTGAC	CCGCTCGAGTCTCCATGGATCTGAACAGG
pTRV2-*ATG7*	CGGAATTCCAAGGAGGGAGGAATCATCA	CCGCTCGAGATGTCCAGGCATCGGAATAG

Table S2. Gene-specific primers designed for qRT-PCR.

Gene	Accession numbers	Forward primer	Reverse primer
actin	AB199316	TGGTCGGAATGGGACAGAAG	CTCAGTCAGGAGAACAGGGT
2-CP1	XM004249364	GAGCTGGAGGGAGAGAACAG	GTGGCTCAAGGGACAAATCT
2-CP2	XM004228521	CCTGCTTCGAGTTTTGAG	GTTTTAAGAACCATCCTTTT
CAT2	NM001247257	AGGAGAATTGGAGGGTCCTT	TGTGAATGTGTGGACACCAG
sAPX	EU251405	GAGGTGGAGCTAATGGAAGC	ACTGGCCAGCTGGAATAAAT
tAPX	NM001247702	CCAGACCAGAGCGTAG	GTCCATCTTTCGTGTAT
MDAR	DQ665255	TCCGAACAAACATACCTGGA	CGTGTGTGCAGTTAGCAATG
DHAR2	NM001247295	AGTATGCGTCAAACAAT	ACCTTCAGGGCTTATC
GR	NM001247314	TTGGATGGAACTATGAGGCA	CAGAGTGACATCCGCATTCT
GME1	NM001247805	TGGAGGGTTTATCGCTTCTC	ACCCTAAGATCCGCAAGATG
GME2	NM001247660	TCAGCTTTGATGGCAAGAAC	CCTCAACCCATCCTTCAACT
VTC2/5	NM001279216	CTGATGAAATTGCTGCTCGT	TACAGTGGCAGCTTTGAACC
GLDH	NM001247674	CCGCCACGTATTATTCCTTT	AACCTCATGAGTCCCACTCC
ATG3	XM004240713	GAGAGGAGTTGAACCCGAAG	CGAAGGAAGTTGACAGCAAA
ATG5	XM004231577	AAGGAGGAGGAACTGAAGCA	ACCCAATTCGAGGAGCTAAA
ATG6	NM001247064	CCCATGCAGTCAAACAATTC	CCCTCATGCATTCAAGACAC
ATG7	XM004251048	ATTCAACGGCTAACCGTAC	CAAACTCAGCTTTGGCACAT
ATG9	XM004237133	ATGTGCATCCTGAAATCGAA	GCCTCTCGAAGAACAAGTCC
ATG8a	NM001247701	ACCGGTGATTGTTGAGAAGG	GCGCTGAGCTTAATCCTCTT
ATG8f	NM001247705	TGAGGCTGCTAGGATTAGGG	GCGAATGACATAGACAAATTGC
ATG8h	NM001247710	TTATCCACATTCTGAGTGGCA	TGTTGTTTGAGGCAAGGTGT
ATG10	XM004246945	GGAGAACCCTTGGCAATAGA	TAGTCCCACATGGATGCAAT
